# ACSC Indicator: testing reliability for hypertension

**DOI:** 10.1186/s12911-017-0487-4

**Published:** 2017-06-26

**Authors:** Robin L. Walker, William A. Ghali, Guanmin Chen, Tej K. Khalsa, Birinder K. Mangat, Norm R. C. Campbell, Elijah Dixon, Doreen Rabi, Nathalie Jette, Robyn Dhanoa, Hude Quan

**Affiliations:** 1 Department of Community Health Sciences, University of Calgary, 3280 Hospital Dr. NW, Calgary, AB T2N 4Z6 Canada; 2 O’Brien Institute for Public Health, University of Calgary, Calgary, Canada; 3 Department of Medicine, University of Calgary, Calgary, Canada; 4 Department of Physiology and Pharmacology, University of Calgary, Calgary, Canada; 5 Department of Surgery, University of Calgary, Calgary, Canada; 6 Department of Clinical Neurosciences, Hotchkiss Brain Institute, University of Calgary, Calgary, Canada; 7 Faculty of Nursing, University of Calgary, Calgary, Canada

**Keywords:** Ambulatory care sensitive conditions, Reliability, Quality indicator, Primary care, Avoidable hospitalizations

## Abstract

**Background:**

With high-quality community-based primary care, hospitalizations for ambulatory care sensitive conditions (ACSC) are considered avoidable. The purpose of this study was to test the inter-physician reliability of judgments of avoidable hospitalizations for one ACSC, uncomplicated hypertension, derived from medical chart review.

**Methods:**

We applied the Canadian Institute for Health Information’s case definition to obtain a random sample of patients who had an ACSC hospitalization for uncomplicated hypertension in Calgary, Alberta. Medical chart review was conducted by three experienced internal medicine specialists. Implicit methods were used to judge avoidability of hospitalization using a validated 5-point scale.

**Results:**

There was poor agreement among three physicians raters when judging the avoidability of 82 ACSC hospitalizations for uncomplicated hypertension (*κ* = 0.092). The *κ* also remained low when assessing agreement between raters 1 and 3 (*κ* = 0.092), but the *κ* was lower (less than chance agreement) for raters 1 and 2 (*κ* = -0.119) and raters 2 and 3 (*κ* = -0.008). When the 5-point scale was dichotomized, there was fair agreement among three raters (*κ* = 0.217). The proportion of ACSC hospitalizations for uncomplicated hypertension that were rated as avoidable was 32.9%, 6.1% and 26.8% for raters 1, 2, and 3, respectively.

**Conclusions:**

This study found a low proportion of ACSC hospitalization were rated as avoidable, with poor to fair agreement of judgment between physician raters. This suggests that the validity and utility of this health indicator is questionable. It points to a need to abandon the use of ACSC entirely; or alternatively to work on the development of explicit criteria for judging avoidability of hospitalization for ACSC such as hypertension.

**Electronic supplementary material:**

The online version of this article (doi:10.1186/s12911-017-0487-4) contains supplementary material, which is available to authorized users.

## Background

Internationally, there is widespread interest in the promise of ambulatory care sensitive conditions (ACSC). ACSC are chronic conditions that should be effectively managed in the community with appropriate medical screening, monitoring and follow-up.

Health care leaders are driven to reduce waste and inefficiency through eliminating unnecessary hospital admissions. Reducing avoidable hospitalizations is a leading healthcare reform in many provinces across Canada and internationally [1, 2]. Avoidable hospitalizations are a common and costly occurrence and policymakers have identified them as a quick and easy way to curb healthcare costs. In Canada, initiatives reducing ACSC are often referred to as "right care in the right place at the right time” [3–6].

Hospitalization rates for ACSC have been used in many jurisdictions as a proxy for the presence or absence of appropriate primary and preventive care; more physician visits within a community should result in fewer hospitalizations for ACSC [7]. A lower rate of ACSC is often interpreted as being indicative of better access to primary care, or better quality of primary care services, resulting in prevention of hospitalizations. As a healthcare system performance indicator, ACSC have been used to inform health policy by assessing the primary health care system (including funding models), improving our understanding of the factors that influence health and by identifying gaps in health status and outcomes for specific populations [8]. In Canada, individuals who experience an ACSC hospitalization represent 6.0% of all hospitalized Canadians, and account for nearly 11.0% of all hospital days [9]. However, the variation in ACSC rates across provinces or regions could be caused by true quality of care differences, differences in data quality or other social factors.

Initially ACSC were developed using hospitalization data to identify hospitalizations in the general United States population that could potentially have been avoided with adequate access to appropriate ambulatory or primary care in the community [8, 10]. Since their inception in 1993, hospitalization rates for ACSC have provided the basis for measuring adequacy of ambulatory and primary health care performance in many regions and countries [11–15]. In its national health indicators report, the Canadian Institute for Health Information (CIHI) reports age-standardized hospitalization rates for seven ACSC: angina, asthma, chronic obstructive pulmonary disease, diabetes, epilepsy, heart failure, and hypertension.

Administrative hospital discharge abstract database (DAD) is the only data source for producing and reporting national and provincial rates of ACSC in Canada. Reasons for the widespread use of ACSC are that the DAD is ready to be analyzed and has wide geographic coverage. The DAD is generated by trained medical coders and includes information about all patients admitted to hospital. Each discharge record contains a unique identification number for each admission, location of residence, physician specialty, up to 25 coded diagnoses recorded using the International Classification of Disease 10^th^ Revision﻿, Canadian Modification(ICD-10-CA) coding system, and an indicator flagging the occurrence of death during a hospitalization. To identify ACSC hospitalizations for each of the seven ACSC conditions, CIHI searches the most responsible diagnosis coding field in the DAD using specific ICD codes (e.g. ICD-10-CA codes I10.0, I10.1, I11 for uncomplicated hypertension) [16]. Exclusion criteria include those who died before discharge, individuals aged 75 years and older and admissions recorded as newborn or stillbirth.

Despite widespread use, it is unclear if ACSC is a reliable and valid indicator that accurately measures the performance of the primary health care system. ACSC hospitalization rates can potentially be used as a marker of access to and quality of primary care, however, only if a significant proportion of them are deemed avoidable and judgments of avoidability have high inter-rater reliability (i.e. judgments of avoidability are reproducible). Thus, the reliability of physician judgment of avoidable hospitalizations for ACSC derived from medical chart review needs to be evaluated. If the ratings of avoidable hospitalizations cannot be reproduced reliably between physicians, the validity and utility of this health indicator are questionable. If assessments of avoidable hospitalizations for ACSC derived from medical chart review are (reproducible) reliable between-physician, it will provide a methodological foundation for determining the proportion of ACSC hospitalizations that are truly avoidable based on medical charts. Thus, the purpose of this research was to test the inter-physician reliability (reproducibility) of judgments of avoidable hospitalizations for one ACSC, uncomplicated hypertension, derived from medical chart review. We chose hypertension as it is a highly prevalent condition affecting more than 20% of Canadians [17], the number one reason for primary care physician visits in Canada [18], and the management of hypertension and related complications is estimated to consume 10% of all health care spending [19, 20].

## Methods

### Data sources

We used administrative health data, specifically the DAD from the province of Alberta, Canada from fiscal years 2012/13 to 2014/15. The DAD is generated by trained coders and includes information about all patients admitted to hospital primarily for monitoring Canadian national health services utilization. Each DAD record contains a unique identification number for each admission, a patient chart number, date of admission, date of discharge, location of residence, physician specialty, diagnoses (up to 25 coded diagnoses are recorded using the International Disease Classification 10^th^ Revision, Canadian Modification (ICD-10-CA) coding system), procedures (up to 20), and an indicator flagging the occurrence of death during a hospitalization. The DAD also has a ‘diagnosis-type’ indicator. The coders assign a one digit ‘diagnosis-type’ code to specify the timing of diagnosis. Type M is the most responsible diagnosis, which is defined in Canada as the condition responsible for the greatest resource use during the hospital stay [21].

### Identifying ACSC hospitalization for uncomplicated hypertension in the major diagnosis field

Following CIHI methods [22] (see Additional file 1) we identified ACSC hospitalizations for uncomplicated hypertension (i.e. ICD-10-CA code I10.0 benign hypertension, I10.1 malignant hypertension, and I11 hypertensive heart disease excluding cases with cardiac procedures) between April 1, 2012 and Aug 1, 2014 (fiscal years 2012 to 2014). In the DAD, specifically, we identified ACSC hospitalizations by searching the most responsible diagnosis coding field using ICD-10 codes I10.0x, I10.1x, I11.x and then excluded patients who underwent cardiac procedure, died in hospital, or those younger than age 20 or older than age 75 at the time of hospitalization. If a patient experienced more than one ACSC hospitalization in the study period only the first admission was selected.

### Expert medical chart review

In the period of April 1, 2012 and Aug 1, 2014, a total of 238 patients with an ACSC hospitalization for uncomplicated hypertension were identified in the DAD at the Foothills Medical Centre in Calgary, Alberta. A random number was assigned to each patient and the number shorted decently. We selected the first 82 patient medical charts as the sample. To reach an inter-rater agreement of 0.75 among the three raters, 75 charts needed to be reviewed. To ensure stability of the kappa statistic, we selected 82 charts to be reviewed by 3 raters.

Chart review was conducted by three experienced physicians. We chose general internal medicine specialists as they typically have more experience with hypertension-related hospital admissions compared to other specialties (i.e. surgeons). The three physicians routinely worked at hospitals and ambulatory care clinics, and had significant experience admitting patients. All physicians underwent training, including a discussion of data extraction, the definition of complications and avoidability (defined as a hospitalization that might ordinarily have been prevented [23]), and the use of implicit methods for judging avoidability [24]. We did not provide specific diseases for defining vascular complications given that the nature of physician’s judgement is implicit. During training the physician raters reviewed 10 charts together.

The physicians used a questionnaire for the medical chart review that included items to confirm the inclusion and exclusion criteria (see Additional file 2). Once the physicians had gone over all aspects of the chart (including cover page, emergency department notes, narrative summaries, admission notes, consultation reports, surgery/operative reports, physician daily progress notes, physician orders and discharge summaries) they used their professional judgment to implicitly judge the avoidability of the hospitalization using a validated 5-point scale [25–27] (see Table 1). Experts commonly use implicit methods for clinical judgment, which rely on the subjective opinion of the individual judge; no predetermined criteria or factors are used [24]. Implicit methods have traditionally been used to rate avoidability of hospital readmissions [28] and are also commonly used during medical panel ratings or Delphi processes [29–32].

**Table 1:** Instruction and validated 5-point scale given to physician raters to judge avoidability of ACSC* hospital admission for uncomplicated hypertension, using implicit judgment

After due consideration of the clinical details of the patient’s medical chart, rate, on a 5-point scale, your confidence in the evidence for avoidability of the hospitalization:
1. Virtually no evidence of avoidability of hypertension admission
2. Slight to modest evidence of avoidability of hypertension admission
3. Evidence of avoidability of hypertension admission is a “close call” (50/50)
4. Strong evidence of avoidability of hypertension admission
5. Virtually certain evidence of avoidability of hypertension admission

*ACSC ambulatory care sensitive condition

### ACSC hospitalization for uncomplicated hypertension in coding fields 2 to 25

In addition to searching the major diagnosis field for uncomplicated hypertension as reason for admission as per CIHI’s methodology, we search the secondary diagnosis coding fields (2 to 25) in the DAD using ICD-10 codes I10.0x, I10.1x, I11.x between April 1, 2012 and Aug 1, 2014. We did this to assess coding misclassification of uncomplicated hypertension (i.e. non-coding of uncomplicated hypertension in the major diagnosis code). In Canada, the most responsible diagnosis coding field is defined as the condition responsible for the greatest resource use during the hospital stay whereas in the United States it is defined as the reason for hospital admission [21]. Thus, searching secondary diagnosis coding fields allows us to determine if patients were admitted to hospital for hypertension and had a subsequent condition such as pneumonia that resulted in long length of stay at hospital. For those patients the pneumonia was likely to be coded as the most responsible condition and uncomplicated hypertension is likely to be coded as secondary diagnosis.

A total of 12,917 patients with a secondary diagnosis of uncomplicated hypertension were identified in the DAD at the Foothills Medical Centre in Calgary, Alberta. A random sample of 320 medical charts were selected and reviewed by 2 trained nurses. The nurses reviewed the discharge summaries. If uncomplicated hypertension was mentioned or listed as a most responsible diagnosis, the chart was flagged. All of the flagged charts were then reviewed by the same three physicians as per the methodology described above.

### Other study variables

The DAD was used to determine all other study variables: age, sex, income quintile, region, and Charlson Comorbidity. A measure of socioeconomic status and income quintile was assigned using the 2001 Statistics Canada Census data. Specifically, median household income for each Census dissemination area, the smallest geographic unit for which Census data are released, was linked to each patient’s postal code assigned using the Statistics Canada Postal Code Conversion file [33] Rural and urban status was also defined using postal code. Charlson comorbidities [34] were derived from the DAD and three categories of comorbidity were considered: (1) no comorbidity; (2) vascular risk-related comorbidity (myocardial infarction, congestive heart failure, peripheral vascular disease, cerebrovascular disease, diabetes with and without complications and renal disease); and (3) unrelated comorbidity (ie, unrelated to vascular risk including dementia, chronic pulmonary obstructive disease, connective tissue disease, rheumatic disease, peptic ulcer disease, paraplegia/hemiplegia, liver disease, cancer/metastatic carcinoma, and AIDS/HIV).

### Statistical analysis

Reproducibility on the 5-point scale across raters was assessed. The reason for using the scale is that avoidable hospitalization is determined by multiple factors, including disease severity (such as acuity of disease), family support to patients, self-management of disease, healthcare system or continuity of care planning (from primary care to tertiary care and vice versa), geographic location, and socio-economic status [35]. There are no objective clinical criteria to determine appropriateness of admission. Then, the scale was dichotomized and assessed using the following cut point: not-avoidable points 1-3 and avoidable points 4-5 for easy interpretation. Fleiss’s kappa statistics for the 5-point scale and Cohen’s kappa statistic (*κ*) for the dichotomized variable and their *p*-value were used to assess agreement among the physician raters [36]. The *κ* was interpreted as follows: a near perfect agreement (0.81 ≤ *κ* < 1.0), substantial agreement (0.61 ≤ *κ* < 0.80), moderate agreement (0.41 ≤ *κ* < 0.60), fair agreement (0.21 ≤ *κ* < 0.40), slight agreement (0.01 ≤ *κ* < 0.20), and less than chance agreement (*κ* < 0) [37]. Analyses were performed with SAS statistical software version 9.3 (SAS Institute Inc, Cary, North Carolina). The institutional ethics review board for the University of Calgary approved the study.

## Results

### ACSC hospitalization for uncomplicated hypertension in the major diagnosis field

In total, 238 ACSC hospital admissions for uncomplicated hypertension were identified in the most responsible diagnosis coding field at the Foothills Medical Centre in fiscal years 2012 to 2014. Among the 82 patients whose medical charts were reviewed, 34.2% were younger than 50, 36.6% were aged 50 to 64 years, and 46.3% were male. Most patients resided in an urban setting (95.1%), and 45.1% had no Charlson comorbidities (Table 2). The average length of stay for an ACSC hospitalization for uncomplicated hypertension was 4.5 days.

**Table 2:** Characteristics of patients with an ACSC* hospitalization for uncomplicated hypertension (aged 20 to 74, fiscal years 2012 to 2014)

	Study Population	
	n	%
Age (years)		
20-49	28	34.2
50-64	30	36.6
65-74	24	29.2
Sex		
Female	44	53.7
Male	38	46.3
Adjusted household income (quintiles)		
1 (lowest)	12	14.6
2	14	17.1
3	9	11.0
4	14	17.0
5 (highest)	15	18.3
Missing	18	22.0
Region		
Rural	4	4.9
Urban	78	95.1
Charlson Comorbidity		
None	37	45.1
Vascular related	43	52.4
Not related	2	2.4

*ACSC ambulatory care sensitive condition

Using the validated 5-point scale, there was poor agreement among the three physician raters when judging the avoidability of ACSC hospitalization (*κ* = 0.092, Table 3). The *κ* remained low level when assessing agreement between raters 1 and 3 (*κ* = 0.092), but the *κ* was lower (less than chance agreement) for raters 1 and 2 (*κ* = -0.119) and raters 2 and 3 (*κ* = -0.008). When the 5-point scale was dichotomized, there was fair agreement among three raters (*κ* = 0.217) and among rater 1 and 3 (*κ* = 0.388). The agreement was very low for raters 2 and 3 (*κ* = 0.157), and raters 1 and 2 (*κ* = -0.009).

**Table 3:** Kappa agreement (and *P*-value) between the three raters by score 1 to 5 and score 1-3, 4-5

Score	1, 2, 3, 4, 5		1-3, 4-5	
Rater	2	3	2	3
1	-0.119 (0.972)*	0.093 (0.057)	-0.009 (0.530)	0.388 (<0.001)
2	-	-0.008 (0.551)	-	0.157 (0.077)
All 3 raters		0.093 (0.057)		0.217 (0.001)

*p-value

**Fig. 1:**
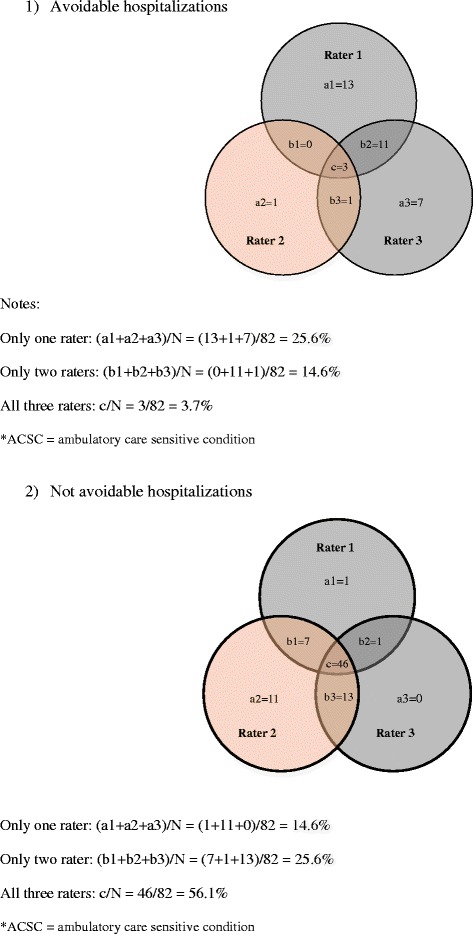
Proportion of ACSC* hospital admission for uncomplicated hypertension rated as 1) avoidable and 2) not avoidable” by only one rater, only two raters and all three raters (fiscal years 2012 to 2014)

The proportion of ACSC hospitalizations for uncomplicated hypertension that were rated as avoidable was 32.9%, 6.1% and 26.8% for raters 1, 2, and 3, respectively (Table 4). The Venn diagram (Fig. 1) describes the agreement between raters, showing that 3 cases (3.7%) were rated as avoidable by all 3 raters, 12 cases (14.6%) by only 2 raters, and 21 cases (25.6%) by only 1 rater. The number of cases rated as *not* avoidable by all 3 reviewers, by only 2 and by only 1 rater was 46 (56.1%), 21 (25.6%) and 12 (14.6%), respectively.

**Table 4:** Proportion of ACSC* hospital admission for uncomplicated hypertension rated as ^a^“avoidable” by patient characteristics (aged 20 to 74, fiscal years 2012 to 2014) and by physician raters

	Rater 1	Rater 2	Rater 3
	n (%)	n (%)	n (%)
Avoidable Hospitalization	27 (32.9)	5 (6.1)	22 (26.8)
Age (years)			
20-49	14 (51.9)	2 (40.0)	12 (54.5)
50-64	5 (18.5)	1 (20.0)	6 (27.3)
65-74	8 (29.6)	2 (40.0)	4 (18.2)
Sex			
Female	13 (48.2)	1 (20.0)	9 (40.9)
Male	14 (51.8)	4 (80.0)	13 (59.1)
Adjusted household income (quintiles)			
1 (lowest)	3 (11.1)	0 (0.0)	1 (4.5)
2	2 (7.4)	1 (20.0)	4 (18.2)
3	4 (14.8)	0 (0.0)	4 (18.2)
4	5 (18.5)	1 (20.0)	4 (18.2)
5 (highest)	8 (29.7)	1(20.0)	5 (22.7)
Missing	5 (18.5)	2 (40.0)	4 (18.2)
Region			
Rural	1 (3.7)	1 (20.0)	1 (4.6)
Urban	26 (96.3)	4 (80.0)	21 (95.4)
Charlson Comorbidity			
None	14 (51.9)	2 (40.0)	9 (40.9)
Vascular related	12 (44.4)	2 (40.0)	12 (54.6)
Not related	1 (3.7)	1 (20.0)	1 (4.5)

*ACSC ambulatory care sensitive condition

^a^avoidable defined as points 4-5 on the 5-point validated scale

### ACSC hospitalization for uncomplicated hypertension in coding fields 2 to 25

There were 12,917 hospital admissions with uncomplicated hypertension listed as a secondary diagnosis (coding fields 2 to 25 in the DAD) at the Foothills Medical Centre and the Peter Lougheed Centre in Calgary, Alberta during the study period. Among the randomly chosen 320 medical charts reviewed by 2 nurses, 14 (4.4%) were flagged as admissions where uncomplicated hypertension was potentially the most responsible diagnosis. After reviewing the medical charts the three physicians agreed that 6 of the 14 (42.8%) admissions were actually ACSC hospital admissions for uncomplicated hypertension. When the 5-point scale was dichotomized, one physician rated one chart as an avoidable hospitalization. All other charts were rated as not avoidable by all three physician raters.

## Discussion

Internationally, ACSC hospitalization rates are being used as a quality-of-care measure. Indeed ACSC admissions may indicate challenges with access to or signal sub-optimal primary care quality, but only if these ACSC admissions are truly avoidable. This study assessed the reliability of specialist ratings of avoidable ACSC hospitalizations for uncomplicated hypertension derived from medical chart review. Importantly, this study found a low proportion of hospitalizations rated as avoidable, and poor to fair agreement of judgment between physician raters. Given that the ratings of avoidability were low and were poorly reproduced, the study findings suggest that the validity (both face and construct) and utility of this health indicator is questionable. Therefore it may be unlikely that ACSC rates would significantly decrease with improved primary care management of uncomplicated hypertension as this health indicator may be too “soft” to measure performance of the primary healthcare system.

Although our study findings question the premise of the ACSC indicator, it may only be true at the level of implicit judgment of medical charts. In implicit review, sometimes termed peer review, the reviewer judges what is being studied (i.e. avoidability of hospitalization) against his/her own clinical knowledge, opinions and beliefs. Thus, implicit review may be biased by reviewers’ experience, consistency, attention to detail, and harshness of judgment [38]. This begs the question: Can multiple physician reviewers judge avoidability of ACSC hospitalizations in the same way based on their own clinical experience? Our findings clearly demonstrate that this is difficult to do, which reflects findings from other studies assessing avoidability of hospital readmissions and quality of care [24, 39–41].

One potential reason for the low ratings of avoidability and the poor to fair agreement of judgment between reviewers may be due to the definition of ACSC; the concept of ACSC is clear (i.e. no need to be hospitalized if the patient receives appropriate out-patient care), but it is hard to operationalize in practice. Another reason is that a medical chart may be insufficient to judge avoidability, which requires comprehensive information such as primary care visits, hypertension management, diagnoses/investigations, drug adherence and patient self-management. When this information is not documented in the medical chart, judging the avoidability of a hospital admission poses a great challenge. In the future, seamless health data exchange between in-patient and out-patient providers will likely provide a more comprehensive resource to assess these important factors. Another potential reason is differing views of the definition of avoidability. For example, one rater could view lack of drug adherence as a patient issue and judge the hospital admission as unavoidable whereas another rater may believe it is amenable to intervention by the physician and hence judge the admission as avoidable. Lastly, there are no clear clinical guidelines for hospital admissions for patients with uncomplicated hypertension, making judgments of avoidability challenging.

One way to improve agreement of judgment of avoidability of ACSC hospitalizations for uncomplicated hypertension would be to develop explicit criteria. Explicit judgments rely on predetermined criteria set by group agreement. Explicit review has a certain “boilerplate” character because the same standards are applied to every case [41] and the burden of accuracy falls on the criteria, not on the reviewer. Unfortunately, such criteria for judging avoidable hospitalization due to ACSC are not available. To increase reproducibility of ratings explicit methods to guide judgment of avoidable hospitalizations based on medical charts (i.e. tools such as a checklist or decision tree) need to be developed.

Currently the DAD is the only data source for producing and reporting on ACSC statistics. In Canada, the most responsible diagnosis coding field is defined as the condition responsible for the greatest resource use during the hospital stay whereas in the United States it is defined as the reason for hospital admission [21]. Thus, we completed a medical chart review where uncomplicated hypertension was listed in the secondary diagnosis field to determine if patients were admitted to hospital for hypertension and had a subsequent complication such as stroke or congestive heart failure. We did this because for those patients the complication was likely to be coded as the most responsible condition. Our findings show that only a small proportion (1.6%) of charts were such cases. Further, it has been shown that coding hypertension in the DAD is done reasonably well. Quan et al. assessed hypertension coding accuracy using ICD-10 and reported sensitivity 68.3%, specificity 97.8%, positive predictive value 93.1%, and negative predictive value 87.7%. Overall it appears that coding misclassification does not significantly affect ACSC hospitalization rates.

Our study has limitations. First, medical chart review was conducted using only hospital medical records as linked hospital and outpatient medical records were not available. Second, we assessed only one of the seven ACSC reported by CIHI. However the ACSC that was chosen, hypertension, is a highly prevalent and high impact chronic condition commonly encountered within all clinical settings and is of major interest from a public health perspective. It is unclear if the results of this study are also applicable to the other ACSC conditions. Third, we reviewed medical charts in Calgary and did not assess for potential differences between tertiary care and community hospitals. Fourth, Canada has very high treatment and control rates for hypertension relative to other countries [17, 42]. Thus, these finding may only relate to Canada. In countries with low control rates of hypertension, the proportion of hospitalizations rated as avoidable may be higher.

## Conclusions

In conclusion, this study found a low proportion of ACSC hospitalizations for uncomplicated hypertension were rated as avoidable. Assessments of avoidability were not reliably reproduced between physician raters, with only poor to fair agreement. These findings either points to a need to abandon the use of the ACSC entirely (since the notion of avoidable hospital stays is so central to the concept of ACSC); or alternatively a need to work on the development of more explicit criteria for judging avoidability (i.e. a Q&A checklist for physician reviewers, or a decision-tree logic model to aid decisions). As it stands currently, the validity and reliability of this health indicator are questionable and it is potentially dangerous to link ACSC hospitalization rates to primary care funding models. As ACSC is a potential tool to trigger positive cases for further exploration, we recommend future research to develop explicit criteria for judging avoidability of ACSC hospitalizations.

## Additional files

Additional file 1: Canadian Institute for Health Information methodology to identify ACSC hospitalizations for uncomplicated hypertension in the discharge abstract database. (DOC 33 kb)

Additional file 2: Questionnaire used by physicians when reviewing medical charts. (DOC 144 kb)

## Abbreviations

ACSC: Ambulatory care sensitive conditions
CIHI: Canadian Institute for Health Information
DAD: Discharge abstract database
ICD-10-CA: International Classification of ﻿Disease﻿﻿ 10^th^ Revision, Canadian Modification
