# Supplementary material for: ACSC Indicator: testing reliability for hypertension
Source: BMC Med Inform Decis Mak. 2017 Jun 26;17:90. doi: 10.1186/s12911-017-0487-4 (PMC5485699; doi:10.1186/s12911-017-0487-4)
Supplement: Supplementary file 1 — Canadian Institute for Health Information methodology to identify ACSC hospitalizations for uncomplicated hypertension in the discharge abstract database. (DOC 33 kb) [file 12911_2017_487_MOESM1_ESM.doc]

**Additional file 1: Canadian Institute for Health Information m**ethodology to identify ACSC hospitalizations for uncomplicated hypertension in the discharge abstract database.

| **Ambulatory Care Sensitive Condition** | **ICD-10-CA Codes in Major Diagnosis Field** | **Exclusion Criteria: Procedure Codes †** | **Other Exclusion Criteria** |
| --- | --- | --- | --- |
| Uncomplicated Hypertension | ICD-10-CA: I10.0*, I10.1*, I11* | 1HA58, 1HA80, 1HA87, 1HB53, 1HB54, 1HB55, 1HB87, 1HD53, 1HD54, 1HD55, 1HH59, 1HH71, 1HJ76, 1HJ82, 1HM57, 1HM78, 1HM80, 1HN71, 1HN80, 1HN87, 1HP76, 1HP78, 1HP80, 1HP82, 1HP83, 1HP87, 1HR71, 1HR80, 1HR84, 1HR87, 1HS80, 1HS90, 1HT80, 1HT89, 1HT90, 1HU80, 1HU90, 1HV80, 1HV90, 1HW78, 1HW79, 1HX71, 1HX78, 1HX79, 1HX80, 1HX83, 1HX86, 1HX87, 1HY85, 1HZ53 rubric (**except** 1HZ53LAKP), 1HZ55 rubric (**except** 1HZ55LAKP), 1HZ56, 1HZ57, 1HZ59, 1HZ80, 1HZ85, 1HZ87, 1IF83, 1IJ50, 1IJ55, 1IJ57, 1IJ76, **1IJ86,** 1IJ80, 1IK57, 1IK80, 1IK87, 1IN84, 1LA84, 1LC84, 1LD84, 1YY5 | - Death before discharge - Individuals age 75 years and older - Admission category recorded as newborn or stillbirth |

Note: ICD=International Classification of Disease, *excluding cases with cardiac procedures, †cardiac procedure codes may be recorded in any position.

*ACSC = ambulatory care sensitive condition
