# Supplementary figures and images for: ACSC Indicator: testing reliability for hypertension
Source: BMC Med Inform Decis Mak. 2017 Jun 26;17:90. doi: 10.1186/s12911-017-0487-4 (PMC5485699; doi:10.1186/s12911-017-0487-4)

**Additional file 2: Questionnaire used by physicians when reviewing medical charts**


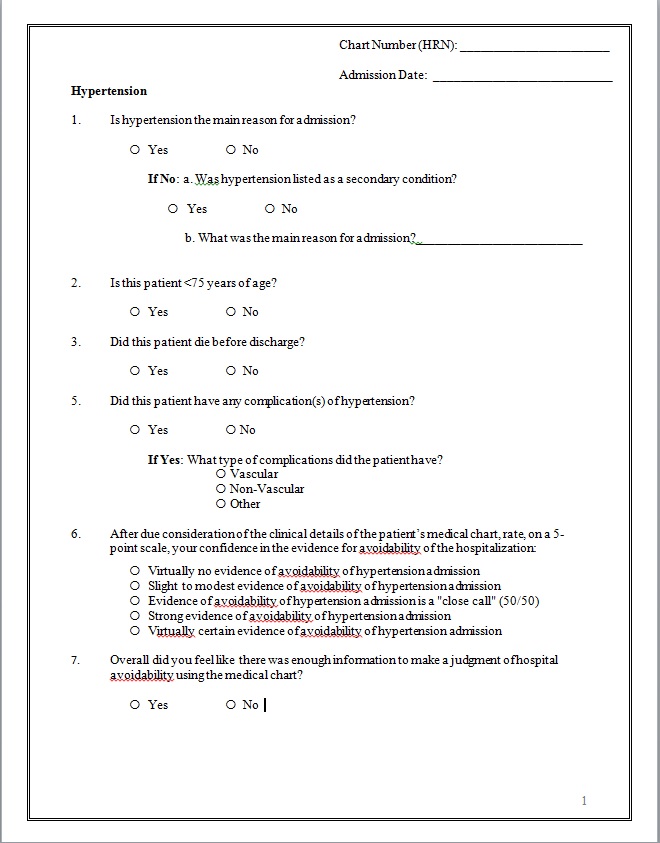

Supplement: Supplementary file 2 — Questionnaire used by physicians when reviewing medical charts. (DOC 144 kb) [file 12911_2017_487_MOESM2_ESM.doc]
